# Determining the ecological security pattern and important ecological regions based on the supply–demand of ecosystem services: A case study of Xuzhou City, China

**DOI:** 10.3389/fpubh.2023.1087588

**Published:** 2023-02-14

**Authors:** Ziyi Wang, Ji Zhang, Jiangchang Chen, Huizhi Gao, Jiaming Li, Muhan Li

**Affiliations:** ^1^School of Geography, Geomatics and Planning, Jiangsu Normal University, Xuzhou, China; ^2^School of Architecture and Urban Planning, Nanjing University, Nanjing, China; ^3^Guangzhou Urban Planning and Design Survey Research Institute, Guangzhou, China; ^4^Institute of Geographic Sciences and Natural Resources Research, Chinese Academy of Sciences, Beijing, China; ^5^School of Architecture, Tianjin University, Tianjin, China

**Keywords:** ecological security pattern, important ecological regions, supply-demand of ecosystem services, circuit theory, research framework

## Abstract

The supply–demand for ecosystem services (ESs) is the bridge between ecological security patterns (ESPs) and human wellbeing. This study proposed a research framework of ESP of “supply–demand–corridor–node” and took Xuzhou, China, as a research case, providing a new perspective for the construction of ESPs. The framework was divided into four sections: identifying the ecological source based on the ESs supply; utilizing multi-source economic-social data to characterize the demand of ESs and constructing a resistance surface; defining the ecological corridor in the study area by employing the Linkage Mapper; and identifying crucial ecological protection/restoration areas along the ecological corridor. The results showed that the area of the supply source of ESs in Xuzhou City is 573.89 km^2^, accounting for 5.19% of the city's total area. The spatial distribution of 105 ecological corridors revealed that there were multiple and dense ecological corridors in the middle of the city, but few in the northwest and southeast. A total of 14 ecological protection areas were located primarily in the south of the urban area, and 10 ecological restoration areas were located primarily in the middle and north of the urban area, with a total area of 4.74 km^2^. The findings of this article will be useful in developing ESPs and determining important ecological protection/restoration areas in Xuzhou, China. The research framework could potentially be used in other areas.

## 1. Introduction

In recent years, global climate change and the growth of human activities have caused a number of problems between people and the natural environment, such as ecological patch fragmentation, biodiversity loss, the heat island effect, and soil erosion (1–3). These problems have seriously threatened regional and national ecological security and slowed down sustainable development. In this situation, ecological security patterns (ESPs) offer a practical way to keep the balance between local environmental protection and economic growth. This has become a hot topic of research around the world, especially in China, which is urbanizing quickly (4).

The ESPs are primarily spatial control methods to coordinate natural ecosystems and socio-economic systems, derived from landscape ecology theories and methodologies (5–7). By identifying the significance of ecological processes and functions in various landscape patches, ESPs can delineate significant ecological areas and build a network system that can maintain the continuity of ecological processes and the integrity of regional ecosystems, preventing the disruption caused by urban growth (8). Several advances have been made in the study of ESPs, including their genesis and development mechanisms (9), design and optimization (10), protection of biodiversity (11), allocation of land to the most productive uses (12), and ecological planning of landscapes (13).

With a growing understanding of natural ecological functions, research on regional ESPs based on ESs has gained prominence in recent years. ESs serve as a link between ecosystems and human welfare. The dynamic process by which ESs flow from ecological systems to human-social systems is constituted by the supply–demand of ESs. The supply–demand for ESs research can improve the ESPs' practical integrity and scientific measurement (14), as well as expand its connotation (10). More and more scholars use ESs supply to identify ecological sources. For example, Li et al. (15) determined the ecological source based on the ESs assessment (carbon storage, water retention, soil retention, and habitat quality) and morphological spatial pattern analysis (MSPA) model; Zhang et al. (16) selected three typical indicators, namely soil conservation, water yield, and carbon fixation, to measure the supply of ESs in the Yellow River basin, and used them as important indicators to identify ecological sources; Wang et al. (17) selected eight indicators, such as water conservation, waste disposal, gas regulation, and climate control, to measure the value of ESs, and took areas with high value of ESs as ecological sources.

Nevertheless, the coupling and coordination between ecosystems and economies/societies are dependent not only on the supply capacity of ESs but also on the human demand for ESs (18). Some research combines the supply and demand of ESs to determine ecological sources. For instance, Zhang et al. emphasized that the ability of ESs demand is the key to identifying ecological sources, so human needs were included in the assessment of ESs in the Beijing-Tianjin-Hebei region of China (19, 20); Jiang et al. (21) identified ecological sources through comprehensive ecological supply–demand ratio by combining the ecological background of the Greater Bay Area of Guangdong, Hong Kong, and Macao in China; Cui et al. (22) analyzed both the ESs supply potential and the human demand potential to determine the ecological source. Nevertheless, the expansion of ESs from the source to other units would be influenced by factors such as accessibility, cost, and the degree of economic and social development, which are frequently reflected in the human demand for ESs. Existing research on the application of ESs demand to construct the resistance surface of ESs flow is limited. Therefore, it is appropriate and important to construct a resistance surface based on the measurement results of the resistance value of ESs demand to reflect the potential for dissipation in the process of ESs delivery.

In addition, the current research concentrates on the development of overall ESPs but disregards the identification of crucial protection/restoration areas. Priority management of key areas can improve the overall function of the regional ecosystem at a lower cost, helping to reach the goal of comprehensive optimization of ecological, economic, and social benefits (23, 24). Important ecological protection areas are generally the core nodes of the regional ESPs, which can effectively conserve the region's most influential flagship species (8, 25). Important ecological restoration areas generally restore damaged ecosystem areas by combining policies and technologies in order to increase the circulation of ecological sources (16, 26). The academic contribution of this study is to construct ESPs that consider the supply–demand for ESs and to precisely identify crucial locations for ecological conservation/restoration around the ESPs. This study has three objectives: establishing a research framework of “supply–demand–corridor–node”; utilizing multi-source economic-social data to characterize the demand of ESs and constructing a resistance surface; and using the circuit theory model to identify the key ecological protection/restoration regions within the study area.

## 2. Study area

**Figure 1:**
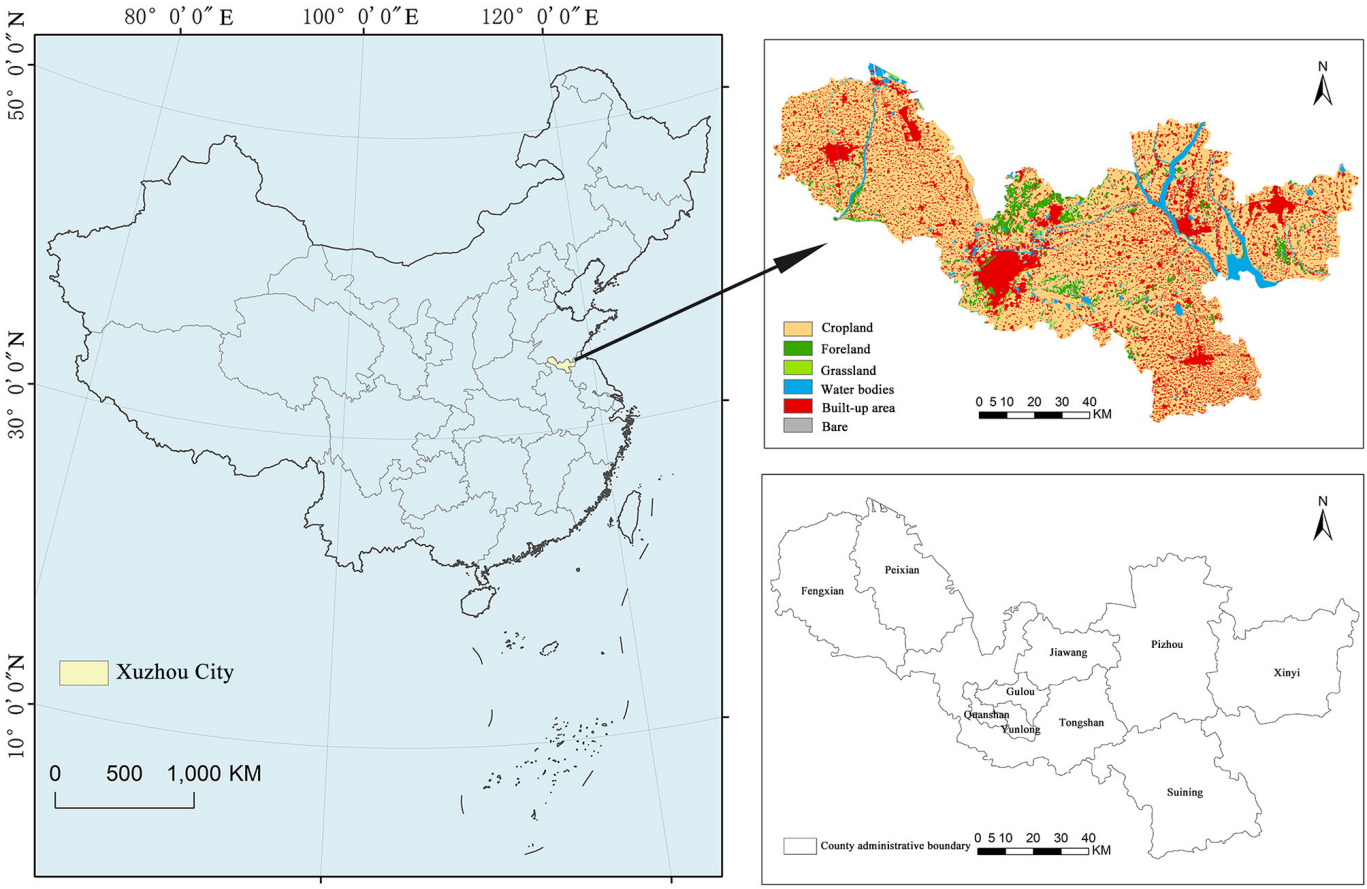
Geographical location and distribution of land cover types in Xuzhou.

Xuzhou is an important node city of China's “Belt and Road” initiative, situated in the northwest of Jiangsu Province between E116°22'−118°40' and N33°43'−34°58', with a total land area of 11,258 km^2^. Except for a few hills in the middle and east, most of the terrain is plain landform. Affected by the temperate monsoon, the annual mean temperature is 14°C and the annual precipitation is 800–930 mm. By the end of 2021, Xuzhou has a permanent population of 9.028 million, with a GDP of 811.744 billion, comprising five districts and five counties (Figure 1). With the rapid development of urbanization and industrialization, Xuzhou is facing the challenge of ecologically sustainable development. Scientific construction of ESPs and accurate identification of ecological restoration/protection areas are of great practical significance for the transformation, development, and ecological restoration of Xuzhou.

## 3. Research framework and data sources

### 3.1. Research framework

**Figure 2:**
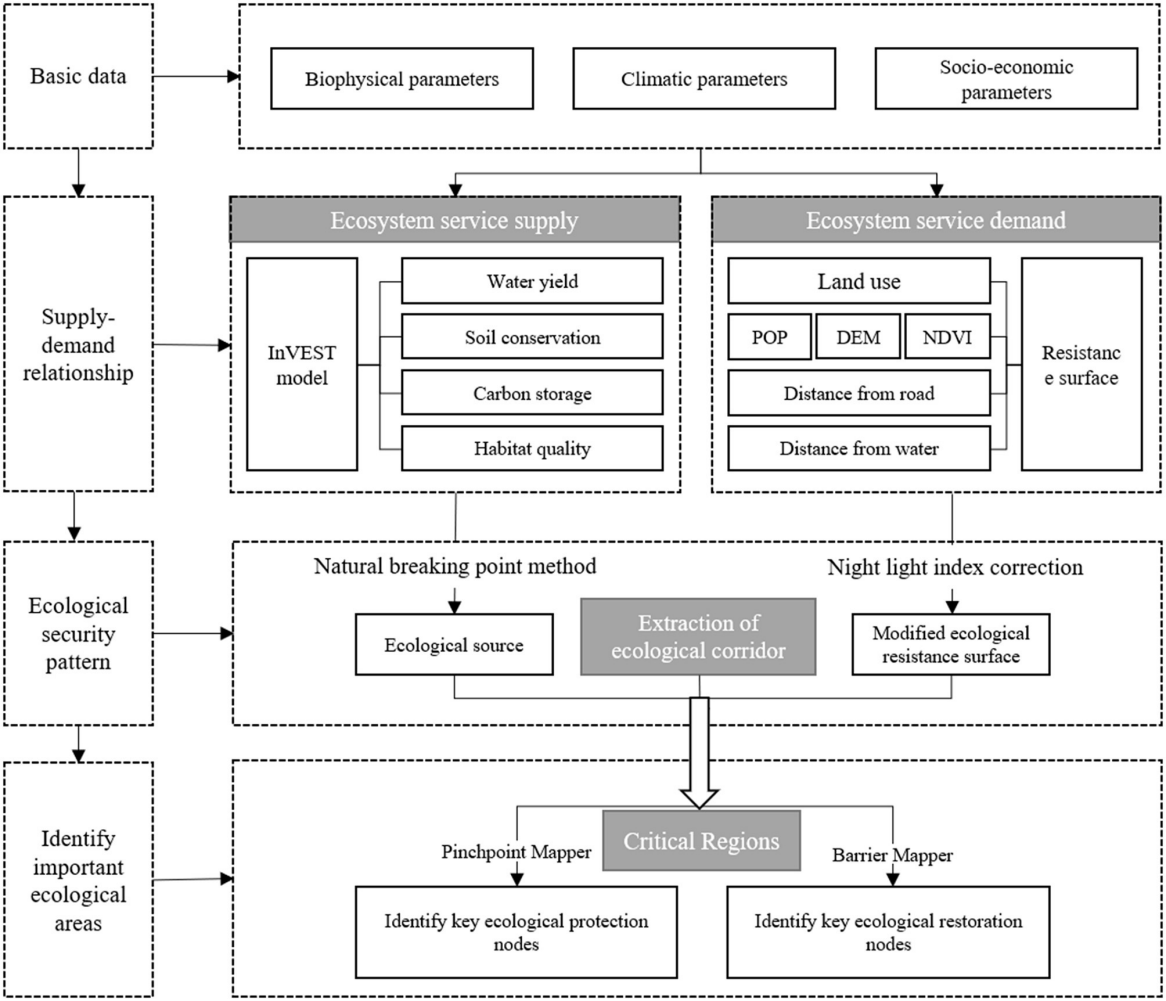
Research framework.

Figure 2 displays the research framework. The ESPs research framework not only helps academics in related fields understand the basic technical logic behind their work but also helps government officials understand how to carry out projects. Based on the supply–demand of ESs, this article designs a research framework of “supply–demand–corridor–node.”

#### 3.1.1. Identify the ecological source through the supply of ESs

In the past, identifying ecological sources was primarily done by directly choosing natural reserves, historical sites, and land use types (27), or by constructing numerical models using pertinent indicators, such as the quantitative ecological importance evaluation system and MSPA (28). Natural ecosystems provide the resources required for human survival and development. As a result, employing ESs supply evaluation to select the ecological source is more in accordance with humanity's actual demands. To acquire the ecological source, we use the InVEST model with four ESs, namely, carbon storage, soil conservation, water yield, and habitat quality, to quantitatively evaluate the ESs supply (29–32).

#### 3.1.2. Construct resistance surface through human demand of ESs

In the past, resistance surface was generated by assigning values to different land use types, superimposing other economic and social factors, and combining the habitat risk assessment model or analytic hierarchy process (AHP) to weigh multiple factors (25, 33). The resistance surface of ESs demand in this article refers to the fact that when ES functions deliver services from an ecological source to other regions, the transmission and transformation of ESs are hampered by the constraints of natural geographical conditions and socio-economic conditions. In essence, it is a potential consumption of ESs.

### 3.1.3. Identify ecological corridors based on the supply—demand of ESs and build ESPs

The ecological corridor is the passage of ecological flow, ecological process, and ecological function in the region. The ecological corridor maintains the stability of the ecological function and ensures the continuity of the ecological process (10, 31). The methods used to build corridors are also diversifying, with the minimum cumulative resistance (MCR) model, the graph theory method, and circuit theory being the prominent examples (34). The MCR model can identify the direction of the biological flow and the best path among them, but it is unable to establish the size of the ecological corridor or the important ecological nodes (3). Circuit theory effectively bridges this gap by simulating biological flow across different resistance surfaces. The size of the current shows how much organisms can move between different patches, and the areas with high current values are important for movement and diffusion (35).

The open-source modeling tool Circuitscape, which is based on circuit theory, determines ecological pinch points and ecological barrier points to represent, respectively, significant areas of ecological protection/restoration. Ecological pinch points are proposed by Mcrae et al. (36) based on circuit theory to represent the key areas that affect the connectivity of the landscape. The identification of ecological pinch points can prevent the degradation or change of ecological sources, so we take ecological pinch points as the key areas of ecological protection. Ecological barrier points are regions where organisms' migration between ecological sources is hindered. Eliminating these spaces will improve communication among ecological sources. This research views ecological barrier points as major areas in need of ecological restoration. Precisely formulate relevant policies and technologies for the identified key protection/repair areas to overcome their internal flaws and external threats. Important area restoration/protection measures are much more effective than those implemented randomly.

### 3.2. Data sources

The main types of data in this study are the administrative scope of the study area, the basic data for calculating the supply of ESs in Xuzhou based on the InVEST model, and the diverse natural and social basic data needed for calculating the demand for ESs in Xuzhou (Table 1). Simultaneously, to maintain data uniformity and correctness, all data are unified to a 30-m spatial resolution under the same coordinate system using Arcgis10.5.

**Table 1:** Data sources.

**Data type**	**Source**	**Explain**
Xuzhou administrative boundary	National Geographic Information Resources Directory Service System (https://www.webmap.cn/commres.do?method=result100W)	The Ministry of Natural Resources gives the national geographic information resources directory service system permission to let people download 1:1,000,000 full-layer elements for free
Land use data of Xuzhou in 2020	Resource and environment science and data center, Chinese Academy of Sciences (http://www.resdc.cn)	The land is put into six groups: cultivated land, forest land, grassland, water area, construction land, and unused land
Digital elevation model	Geospatial data cloud platform (http://www.gscloud.cn)	A solid ground model that shows the height of the ground as a set of ordered arrays of numbers. It is also the basic information you need to figure out the slope
Precipitation data	China Meteorological Data Service Center (http://data.cma.cn)	Using the Kriging interpolation tool, the average annual rainfall can be found
Soil data	Data from global soil data provided in the World Soil Database (HWSD) constructed by the Food and Agriculture Organization of the United Nations (FAO) and the International Institute for Applied Systems (IIASA), Vienna	This data is a grid with a spatial resolution of KM. Each grid point has information about its soil type, soil phase, soil's physical and chemical properties, and more
Potential evapotranspiration data	From CGIAR-CSI GeoPortal (http://www.csi.cgiar.org)	It means how much water can be lost through evaporation from the area covered by plants on the surface below
Demographic and economic data	From the resource and environment science and data center of Chinese Academy of Sciences (http://www.resdc.cn)	Mainly including road traffic data and population density of Xuzhou
NDVI data	From geospatial data cloud (https://www.gscloud.cn)	It is a MOD13Q1 item with a 250 m resolution
Night light data	Adopt NPP-VIIRS data (https://www.ngdc.noaa.gov)	This information is preprocessed and corrected 2020 data that has been algorithmically produced

## 4. Method

### 4.1. Quantifying the supply of ESs

#### 4.1.1. Water yield

Water yield services measure the capacity of an ecosystem to hold rainwater under the combined action of plants and soil. In this article, the Water Production module of the InVEST model was used to obtain the spatial distribution of water production in Xuzhou. Based on the principle of water balance, the module defines water production as the amount of water remaining after subtracting plant transpiration and surface evaporation from the precipitation within the grid. It also assumes that the water production of the grid unit will eventually reach the watershed outlet through the surface and underground runoff. The water production was estimated from parameters such as precipitation, potential evapotranspiration, root system, and soil depth, and finally, the raster water yield of the watershed was calculated. The model's algorithms are as follows:


(1) $$Y_{xj}=\left(1-AET_{xj}/P_{x}\right)\times P_{x}$$

(2) $$\frac{AET(x)}{P(x)}=\frac{1+w_{x}R_{xj}}{1+w_{x}R_{xj}+1/R_{xj}}$$

where *Y*_*xj*_ represents the water yield of the x-th grid of land use type j (m^3^·hm^−2^); AET_xj_ represents the annual actual evapotranspiration of the x-th grid of land use type j (mm); *P*_*x*_ represents the annual average precipitation of the x-th grid (mm); *R*_*xj*_ represents the dryness index of the x-th grid of land use type j; and *w*_*x*_ represents the available water content of vegetation. The details of each coefficient are shown in Supplementary Table A1.

#### 4.1.2. Soil conservation

Soil conservation services are the function of ecosystems to maintain soil by reducing erosion from rainfall through the forest canopy and root system to increase soil erosion resistance, thereby reducing soil erosion and soil loss. The soil in Xuzhou is primarily brown and cinnamon type, with poor water storage conditions and relatively low vegetation coverage (37). It is the key prevention and control area of soil erosion in Jiangsu Province and a plain sand protection area. Therefore, soil conservation is an important indicator to measure the water and soil conservation of the ecosystem in Xuzhou. The SDR module in the InVEST model was utilized in this article to evaluate soil conservation in Xuzhou (26). According to the potential soil loss equation, soil conservation was obtained by reducing actual soil erosion with potential soil erosion as follows:


(3) $$SC_{i}=RKLS_{i}-USLE_{i}$$

(4) $$RKLS_{i}=R_{i}\times K_{i}\times LS_{i}$$

(5) $$USLE_{i}=R_{i}\times K_{i}\times LS_{i}\times C_{i}\times P_{i}$$

where *SC*_*i*_ is soil conservation; *RKLS*_*i*_ is the potential erosion amount; *USLE*_*i*_ is the actual erosion amount; *R*_*i*_ is Rainfall Erosivity Factor; *K*_*i*_ is soil erodibility factor; *LS*_*i*_ is the slope length factor; *C*_*i*_ is the vegetation cover management factor; and *P*_*i*_ is the factor of water and soil conservation measures. The details of each coefficient are shown in Supplementary Table A2.

#### 4.1.3. Carbon storage

The carbon storage service of an ecosystem is the absorption of carbon dioxide from the atmosphere by plants through photosynthesis, which is converted into carbohydrates such as glucose and fixed in the form of organic carbon in the plant or soil. Xuzhou is an example of a historic industrial city in our country. The city's growing industrialization has made it a prominent area for the combustion of chemical fuels. Xuzhou's carbon stock has become an important indicator, affecting the regional climate and the security of the regional ecosystem. In this article, the Carbon module of the InVEST model was used to calculate the amount of carbon stored in Xuzhou, and the amount of carbon currently stored was estimated based on the data of land use types and their corresponding storage in the four carbon pools. The following is the formula:


(6) $$\begin{array}{l} C_{total\;}=C_{above\;}+C_{below\;}+C_{soil\;}+C_{dead\;} \end{array}$$

where C_tota_ is the total carbon storage; C_above_ is aboveground carbon storage; C_below_ refers to underground carbon storage; C_soil_ is soil carbon storage; and C_dead_ is the carbon storage of dead organic matter. The unit is t·hm^−2^. The details of each coefficient are shown in Supplementary Table A3.

#### 4.1.4. Habitat quality

In recent years, heavily urbanized human activities have had an impact on Xuzhou's biodiversity. As a result, habitat quality can evaluate ecosystems' potential to retain and sustain genetic, species, and ecosystem variety, which is the most essential supporting function offered by ecosystems. In this article, the InVEST model's Habitat Quality module was used to assess the biodiversity of the study region and simulate the influence of human activities on the habitat. The more people do, the more the habitat is threatened; the lower the quality of the habitat, and the lower the level of biodiversity. In contrast, the fewer people do, the higher the quality of the habitat and the higher the level of biodiversity. The birth quality is derived by combining the sensitivity of different land use types to threat factors and the degree of external threats as follows:


(7) $$\begin{array}{l} Q_{xj}=H_{j}\left[1-\left(\frac{D_{xj}^{z}}{D_{xj}^{z}+K^{z}}\right)\right] \end{array}$$

where Q_xj_ represents the habitat quality of the x-th patch of land use type J; $D_{xj}^{z}$ represents the habitat stress degree of the x-th patch of land use type j; *H*_*j*_ indicates the habitat suitability; and *K*^*z*^ is the semi-saturation constant. The details of each factor are given in Supplementary Table A4.

The biophysical parameters used in the following model development are mostly drawn from earlier studies (38–40). To eliminate the dimensional effects among the four indicators, the results of the ES assessment were unified and normalized, using the method of natural break point; the four ESs were divided into three grades, namely, extremely important, medium important, and unimportant, specifically, with levels 1 and 2 are low-value zones (not important), levels 3 are medium value zones (medium important), and levels 4 and 5 are high-value zones (extremely important).

### 4.2. Quantifying the demand for ESs

The expansion of ESs from the source to other units is influenced by factors such as accessibility, cost, and the degree of economic and social development, which are often reflected in the human demand for ESs. Therefore, it is reasonable to construct the resistance surface based on the resistance value of ESs demand to reflect the dissipation potential in the process of ESs supply. Due to different types of natural land cover and different levels of human activity interventions, the movement processes of species between regions show differentiated characteristics, including the mobility and transmission of ecological functions. Quantifying the demand for ESs is based on two main perspectives: the natural influences on the generation and transmission of ESs and the degree of demand in human activity areas.

**Figure 3:**
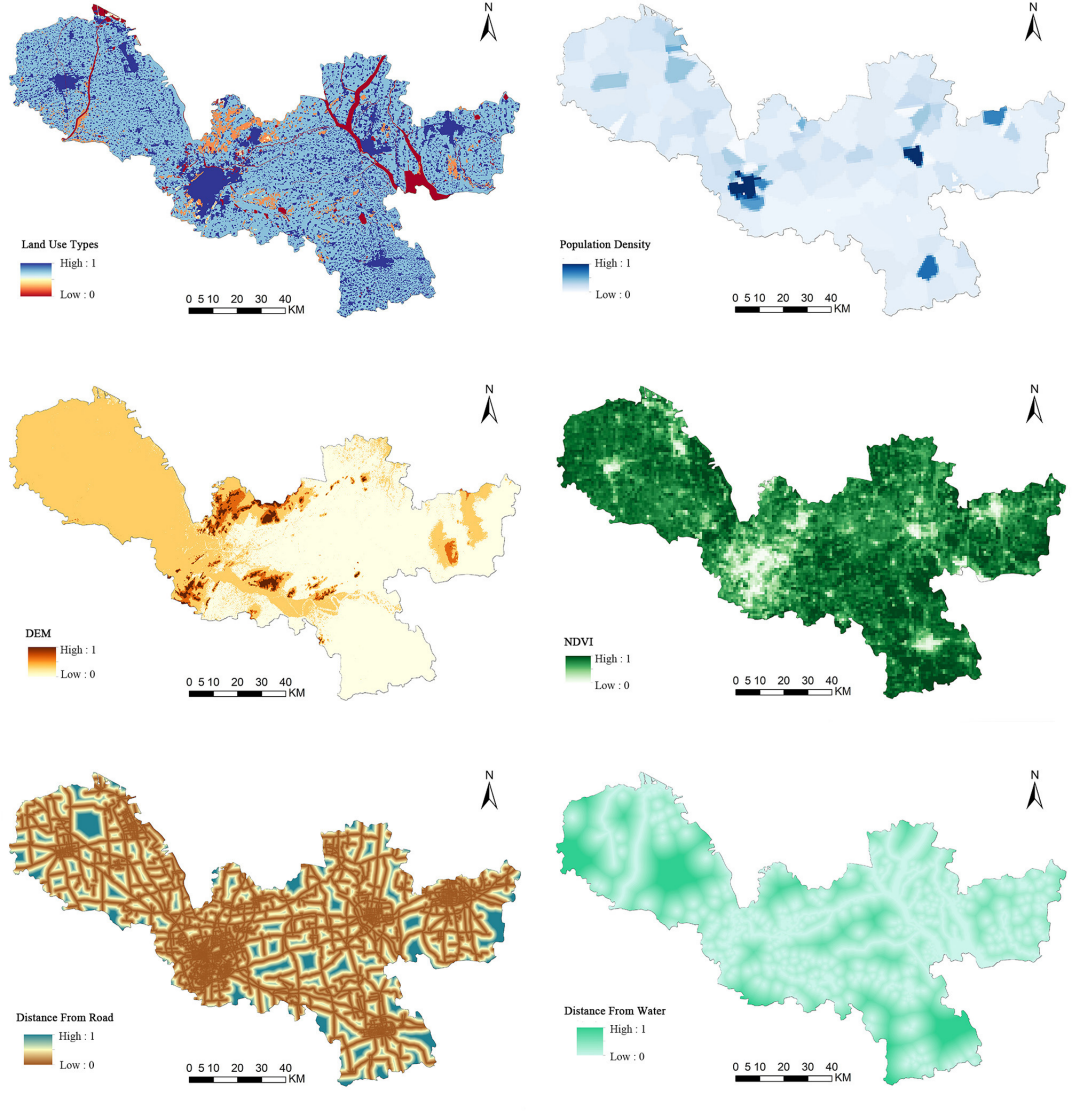
Spatial distribution of ecosystem service demand in Xuzhou.

Referring to previous studies and combining with the natural and economic conditions of the study area (41–45), this article selects six factors, namely, land use types, population density, DEM, NDVI, distance from the road, and distance from water, to characterize human demand of ESs (Table 2). The elevation can determine the flow of ESs by regulating the distribution of water and heat; NDVI can directly affect local climate, biodiversity, soil conservation, and other services; population density, water, and road traffic distribution are important for the development or decline of the region; land use type is a concentrated expression of human activities, which is both an influencing factor and an important carrier of various ESs (Figure 3).

**Table 2:** Resistance factor and its resistance value.

**Resistance indicator**	**Classification**	**Resistance value**	**Weight**
Types of land use	Water	1	0.4
	Woodland	2	
	Grassland	3	
	Cultivated land/bare land	4	
	Construction land	5	
Population density	< 1.42	1	0.2
	1.42–4.74	2	
	4.74–12.10	3	
	12.10–24.20	4	
	>24.20	5	
DEM(m)	< 31	1	0.1
	31–49	2	
	49–78	3	
	78–127	4	
	>127	5	
NDVI	< 0.46	1	0.1
	0.46–0.61	2	
	0.61–0.73	3	
	0.73 – 0.82	4	
	>0.82	5	
Distance to road(m)	< 1,500	1	0.1
	1,546–3,092	2	
	3,092–4,638	3	
	4,638–6,184	4	
	>6,184	5	
Distance to water(m)	< 3,475.68	1	0.1
	3,475.68–6,951.36	2	
	6,951.36–10,427.04	3	
	10,427.04–13,902.71	4	
	>13,902.71	5	

Since human disturbance is one of the important resistances to species dispersal, and nighttime light data can represent the intensity of human activities in the whole region, the higher the value of nighttime light intensity represents the stronger the development vitality of the region (46). Therefore, this article then uses the nighttime light index to modify the ecological resistance coefficient to construct the resistance surface as follows:


(8) $$\begin{array}{l} R^{{}^{*}}=\frac{TLI_{i}}{TLI_{a}}\times R_{o} \end{array}$$

where R^*^ is the corrected grid resistance coefficient; *TLI*_*i*_ is the night light intensity value of grid I; *TLI*_*a*_ is the average night light intensity value of land type a; and *R*_*o*_ is the base resistance value of grid i.

### 4.3. Extraction of an ecological corridor

The circuit theory simulates the migration and diffusion process of species by using the characteristic of the random walk of electrons in the circuit. This scheme can relatively accurately simulate the diffusion path of species in a heterogeneous landscape, even in the absence of species migration data (36). At present, the ecological corridor has been used as a tool to connect the natural ecological source and the needs of human society (44). This article uses the Linkage Pathways Tool module to identify the ecological corridor, which can draw the lowest cost link between core patches according to the core patch and resistance grid. At the same time, the contribution degree of landscape connectivity can be determined through centrality analysis. The greater the centrality of an ecological patch indicates that it can have sufficient material, energy, and information exchange with other patches and that it is easy to establish contact with other patches in the face of disturbance. Therefore, its ability to resist disturbance is relatively strong. The Centrality Mapper tool is used to calculate the flow centrality of the optimal ecological corridor and quantitatively analyze the importance level of the ecological corridor.

### 4.4. Identify ecological protection and restoration nodes

Ecological pinch points represent areas with high-flow density in the ecological network, that is, areas with a high probability of species migration or no alternative path. Priority should be given to protecting such areas during the construction of the ecological network (47). In this article, Pinchpoint Mapper is used to identifying ecological protection areas. Pinchpoint Mapper combines the Circuitscape algorithm with the base map generated by Linkage Mapper to achieve the research purpose. Ecological barrier points refer to areas where the migration process of species between source areas is greatly hindered (48). Taking ecological barrier points as ecological restoration nodes can significantly improve the connectivity between ecological sources. The operation of the Barrier Mapper tool is based on the extraction of ecological corridors by the Linkage Mapper tool. This tool can not only detect the complete obstacle points that hinder the migration of species but also detect the obstacle areas that have some obstacles but do not completely affect the migration of species. This article considers only the areas that have complete obstacles to the migration of species.

## 5. Results

### 5.1. The spatial pattern of supply—demand of ESs

**Figure 4:**
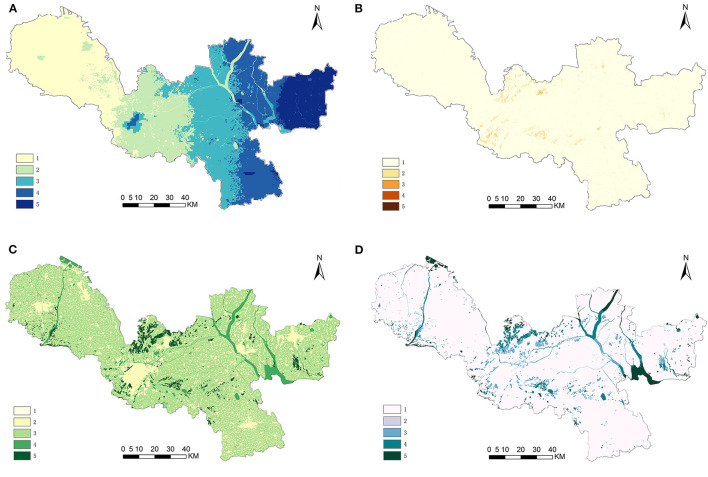
Four types of ESs supply in Xuzhou **(A)** water yield, **(B)** soil conservation, **(C)** carbon storage, and **(D)** habitat quality.

Figure 4 shows that the water yield service decreases gradually from east to west. This was related to the local precipitation, soil properties, and evapotranspiration. The high-value area was distributed in the northeast of Xuzhou City, which was an important water conservation area. Soil conservation services were distributed spatially in Xuzhou in a zonal and dot-overlapping pattern. High-value areas were mainly concentrated in the northern Jiawang District and the peripheral areas of the central urban area, while the rest are mostly low-value areas. Xuzhou's carbon storage service generally presented a pattern of low in the center and high on the edge, high in the north, and low in the south. High-value areas were mainly distributed in Jiawang District in the north of Xuzhou City, Luoma Lake Basin in the east, Dasha River Basin in the northwest, and the green area around the city outside the central urban area. The high-value areas of habitat quality were mainly distributed in areas where forests and lakes were concentrated.

**Figure 5:**
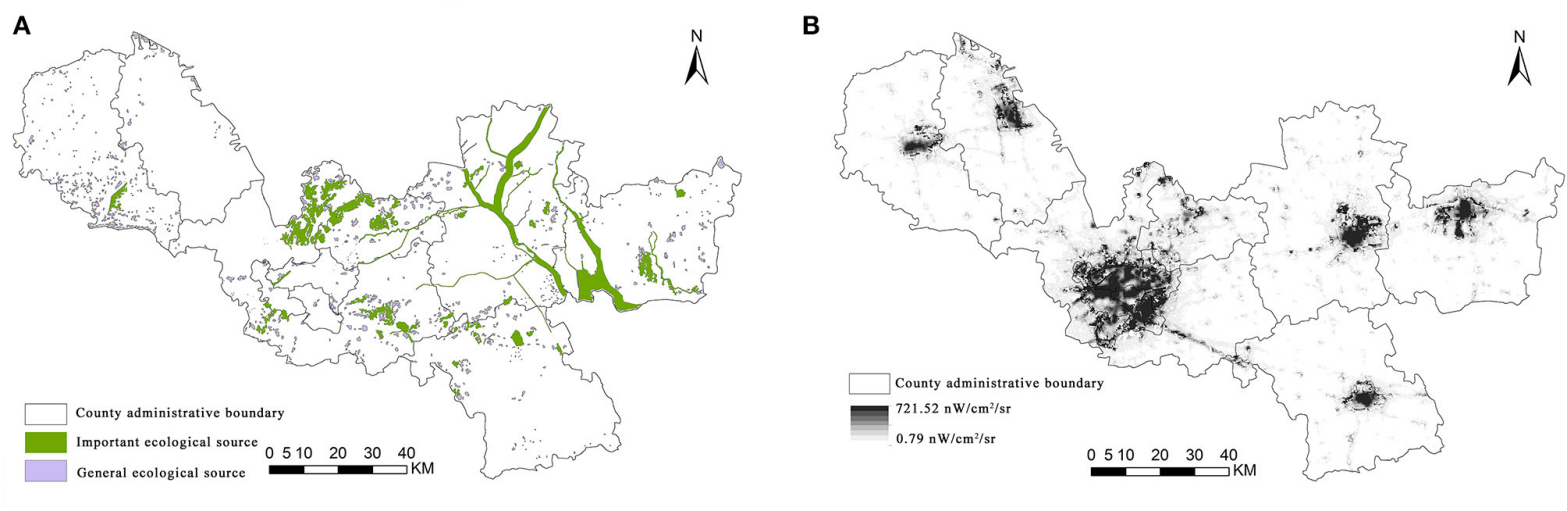
Spatial distribution of ecological sources **(A)** and resistance surface **(B)** in Xuzhou.

A total of 1,107 important ecological sources were extracted from the classification of extremely important regions. If all ecological sources were included in the scope of ESPs, it would lead to fragmentation of the ecological space, which was not conducive to the overall regional optimization strategy. Referring to previous studies (49, 50), this article further extracted patches with an area of more than 0.8 km^2^ as the ESs supply source, representing the main areas with strong ESs formation and supply capacity in Xuzhou. Finally, 47 patches were chosen as ecological sources. The overall area of the ecological source was 573.89 km^2^, accounting for 5.19% of the city's total area. The greatest ecological source patch covered 152.26 km^2^. The ecological source areas, as illustrated in Figure 5A, were concentrated in places where the mountain and water systems are adequately maintained. For instance, the southern part of Fengxian County, the northern part of Jiawang District and Tongshan County, and the Luoma Lake water system in Xinyi. Figure 5B depicted the demand for ESs in Xuzhou, which was identified as a barrier to the supply of ESs. The average ecological resistance was 18.60 nW/cm^2^/sr, with a maximum of 721.52 nW/cm^2^/sr and a low of 0.79 nW/cm^2^/sr. The ecological resistance depicts the collective predicament in space. The ecological resistance represented the predicament of humanity in space. Among them, regions with high resistance values were typically more populated, with a concentration in urban cores such as Quanshan District, Gulou District, Yunlong District, and others. Moreover, urbanized regions such as Fengxian County, Peixian County, Pizhou City, Xinyi City, and Suining County had high resistance values.

### 5.2. Extraction of an ecological corridor

**Figure 6:**
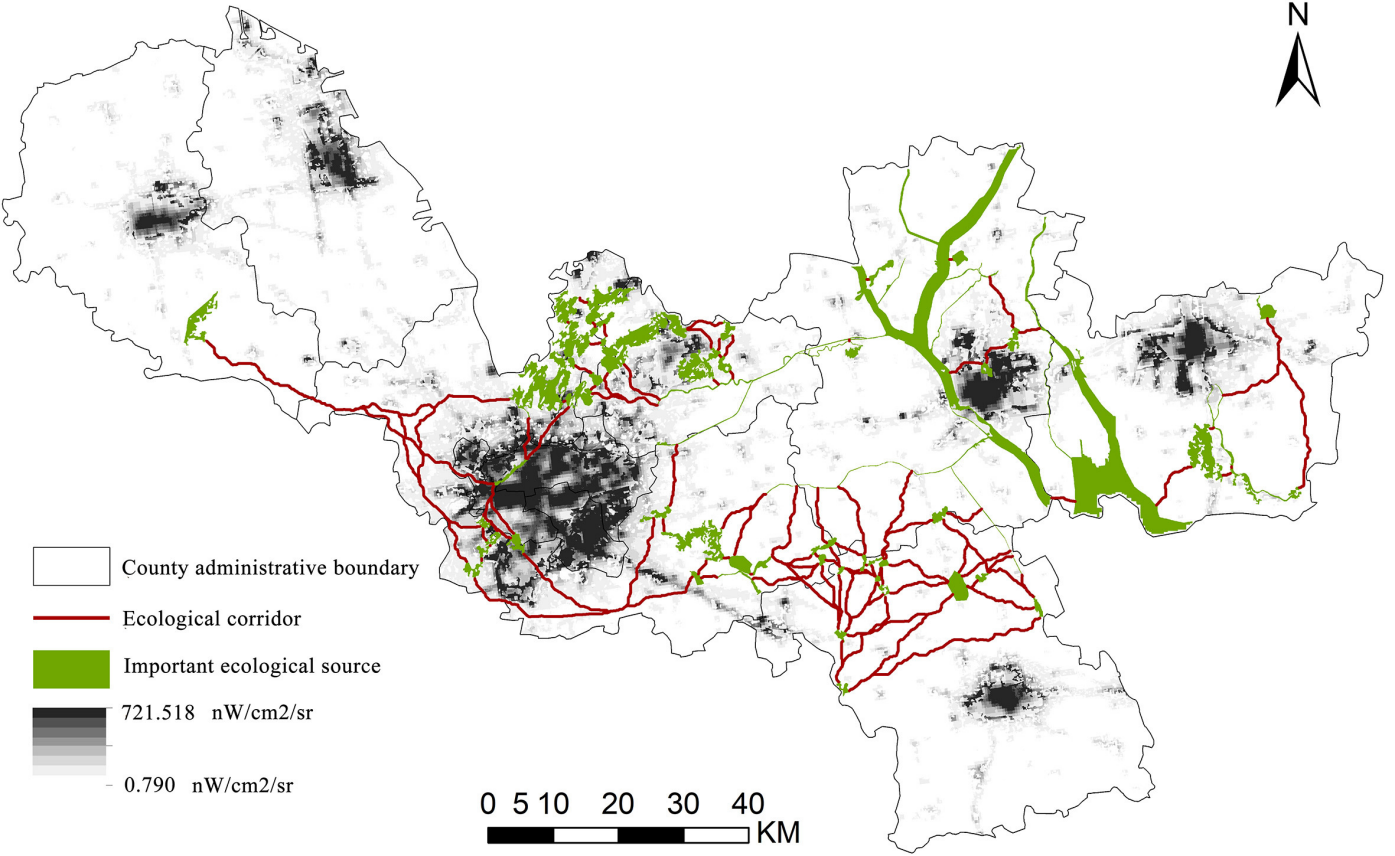
Spatial distribution of ecological corridors in Xuzhou.

The ecological corridor was calculated using Linkage Mapper and superimposed with the study area's source and resistance surface (Figure 6). The ecological corridors in Xuzhou were interconnected in a network, showing that the overall ecological quality was good but connectivity was limited. The article identified 105 ecological corridors that were distributed in a crisscross network. In the central portion of the study area, there were numerous and dense corridors, whereas there were few ecological corridors in the northwest and southeast. The ecological corridors in Xuzhou were overlapped, connected, and blocked each other. At the same time, the ecological corridor had a considerable curve, which could generate a more varied environment and was favorable for increasing the corridor's species richness.

### 5.3. Identification of ecological protection/restoration nodes

**Figure 7:**
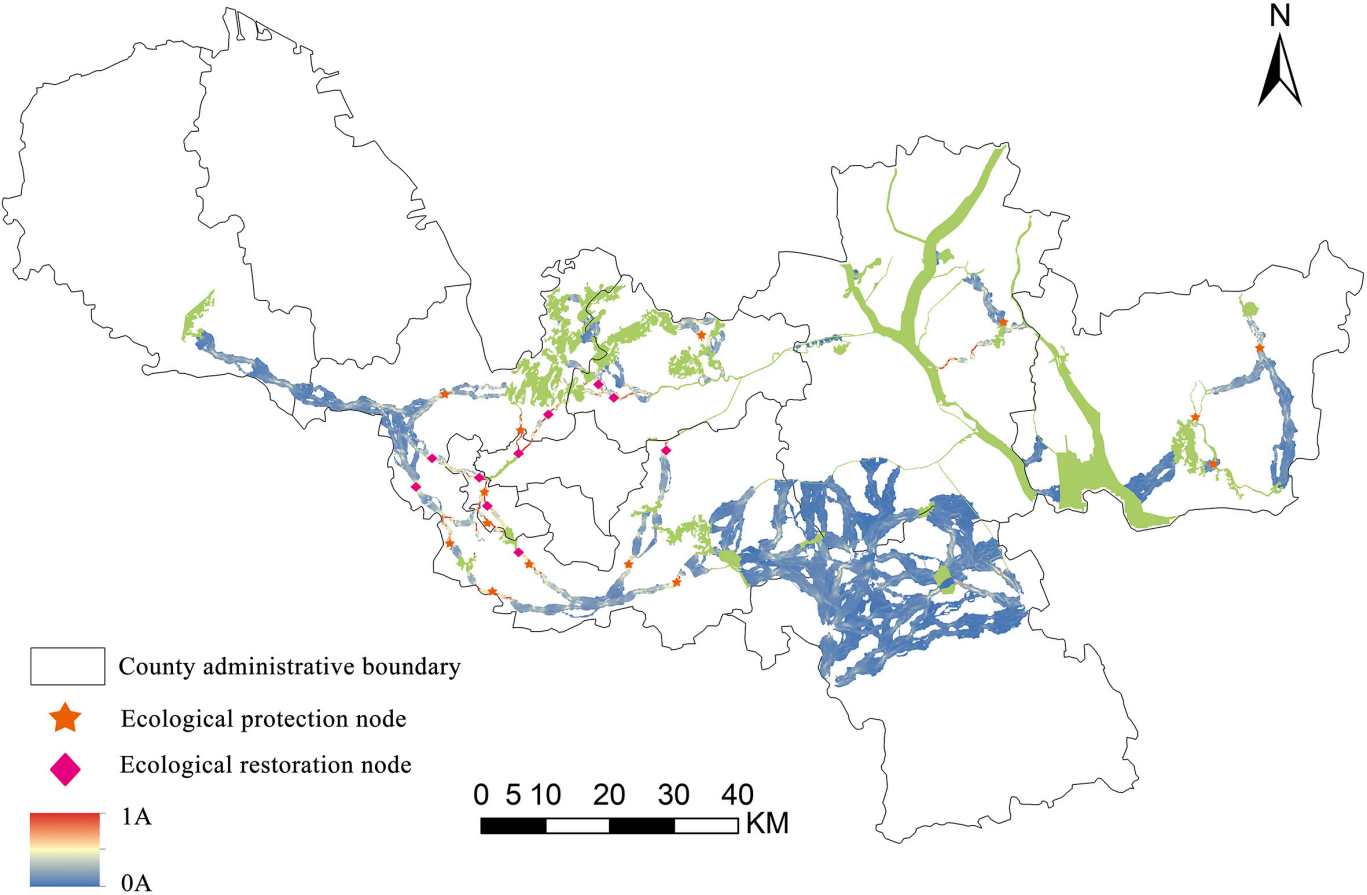
Critical ecological protection/restoration areas in Xuzhou.

On the basis of realizing ecological corridor extraction, the key nodes in the corridor were further identified by combining Pinchpoint Mapper and Barrier Mapper tools (Figure 7). The main urban area's southern region was where 14 significant ecological protection nodes had been found in this article, with a sparse distribution in Jiawang District, Tongshan District, Pizhou City, and Xinyi City. The pinch point was the area in the ecological corridor that needs to be protected. The connectivity between ESs would be impacted if the pinch point's function was lost or damaged. Obstacle points were the places that hindered the connection of ecological patches. The connection between the landscape and ecological stability could be greatly enhanced by the restoration of barrier points. The existing corridor had 10 barriers, and the most of which were found in Tongshan District, Quanshan District, and Gulou District. Key ecological protection/restoration areas in Xuzhou City covered a total area of 4.74 km^2^.

## 6. Discussion

### 6.1. The relationship between supply—demand of ESs and ESPs

Ecological security is the manifestation of ESs, and the supply–demand of ESs is the bridge between ecological security and human wellbeing. Ecological security is an abstract concept that refers to the ecological environment in which humans live and develop being entirely or partially free of risks and having the capacity to address important ecological issues. The objective of the creation of the ESP is to ensure the survival requirements of human society and to realize the sustainable growth of humans and the ecological environment. Its evaluation is centered on the stress and reaction between the ecosystem and human health. The supply–demand of ESs also emphasizes the relationship between the ecosystem and human wellbeing, which is the various benefits that mankind obtains from the ecosystem. Ecological security, on the one hand, is a representation of ESs. ESs supply will fail as a result of economic and social development harming the ecological environment, which will reduce human demand for ESs. For example, due to the long-term discharge of agricultural, industrial, and domestic sewage along the coast of China's Taihu Lake, the breakout of the blue-green algae crisis in Taihu Lake in 2007 directly resulted in a water shortage of millions of people. On the other hand, the balance between the supply and demand of ESs is the link between ecological security and human wellbeing. For instance, urban heat island, air pollution, and other problems are the result of an imbalance between the supply and demand of ESs, which has severely impacted the human and ecological environment's sustainable growth.

The introduction of the perspective of supply and demand for ESs provides crucial theoretical guidance for the development of ESPs. Internationally, this article seems to be the first time to use ecosystem service demand to build the resistance surface of ESP. The supply of ESs from the source to other units is influenced by factors such as accessibility, cost, and the level of economic and social development, which are frequently reflected in human demand for ecological services. This concept offers novel insights for the construction of ESPs.

### 6.2. Influence of resistance threshold on the ecological corridor

The main function of an ecological corridor is to connect different ecological source areas to maintain ecological processes. The spatial scope of the corridor directly affects the supply and demand of ESs (31, 51). The extent of an ecological corridor can be identified by cumulative resistance with a specific threshold, but there is still no consensus on the best approach (8). The distribution of ecological corridors in the study area showed spatial heterogeneity. In the areas with little change of slope and elevation and little human influence, the resistance of species migration and energy flow is small, and the spatial range of the ecological corridor is long. In contrast, in cultivated land and construction areas with high slope, high altitude or more man-made impacts, it is difficult to have appropriate circulation channels for ecological elements (10).

**Figure 8:**
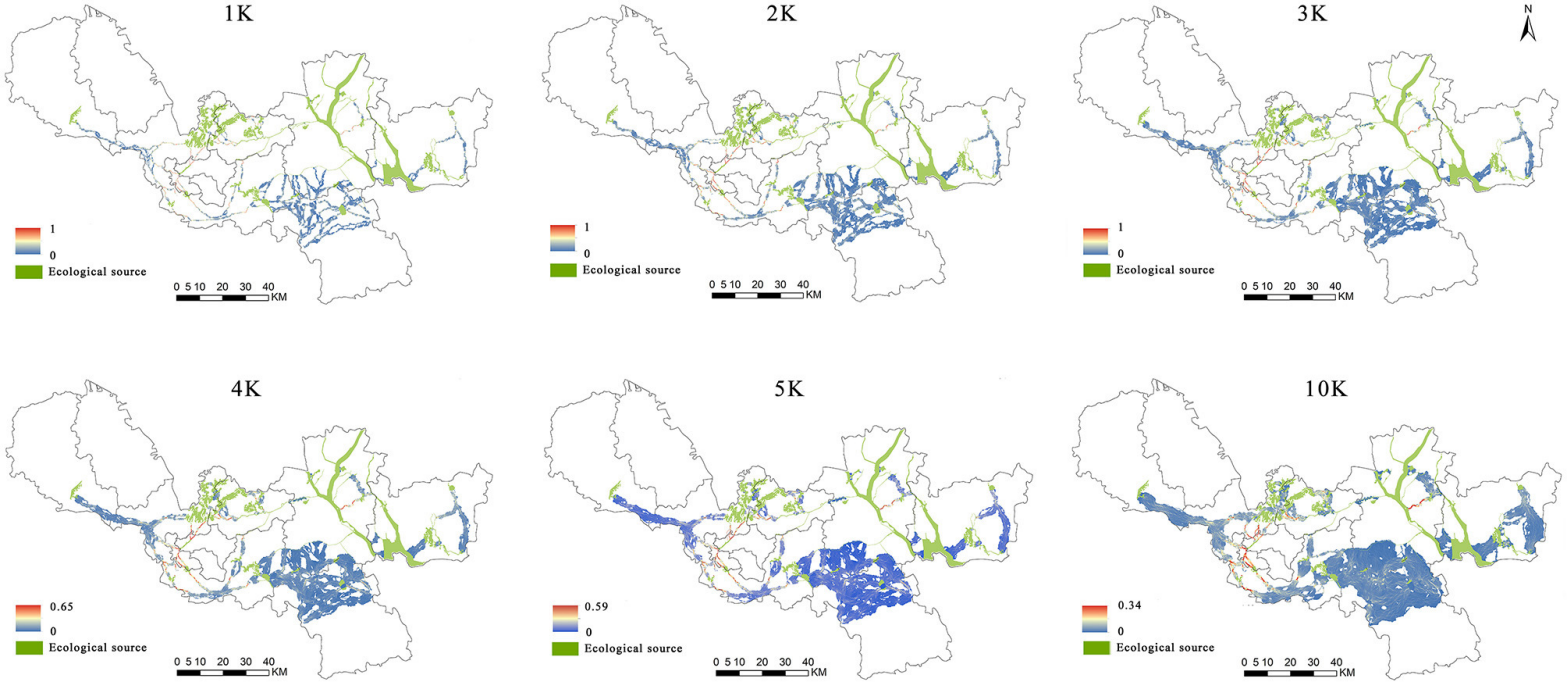
Ecological corridor extent and cumulative proportion at different thresholds (K for thousands and the number in each image represents the distance threshold for corridor recognition).

The ecological function of a corridor is closely related to its spatial scope, which plays a significant part in maintaining ecology. The corridor range distribution was examined in this study using various resistance thresholds, which revealed identical spatial distribution and various width ranges (52). This study attempted to define the spatial extent of the ecological corridor by raising the cumulative resistance threshold from 1 to 10 K (Figure 8). Although the total area of the ecological corridor has increased, the location of pinch points and obstacle points has not changed significantly, indicating that the model is effective for the judgment of the protection/restoration area (16). The larger the threshold value is set, the wider the ecological protection/restoration area will be, and the more expensive the actual protection and restoration will be. This study considers that Xuzhou has a large urban area and a limited budget for ecological protection/restoration. At the same time, under the control of territorial space planning, the oversized ecological network will encroach on a large number of permanent basic farmland and existing construction space, resulting in great difficulty in coordination and thus reducing operability. It is assumed that the funds and policies for ecological protection and restoration can only support 20% of the whole research area (16, 31). Therefore, 3 K is selected as the threshold range.

### 6.3. Policy suggestions on crucial regions of ecological protection/restoration in Xuzhou

Our overall approach to the ecological protection/restoration proposal in Xuzhou is to take into account the supply and demand of ESs, focus on corridor connectivity, and carry out ecological conservation/restoration at key nodes. This is because the most important function of ecological protection or restoration nodes is to be able to achieve focus on key locations within ecologically fragile areas. Combined with the research results, we found that ecological protection nodes are concentrated in the inter-patch corridor and the internal corridor in the south of Jiawang District and Tongshan District, while ecological restoration nodes are mainly distributed in Quanshan District and Gulou District, mostly concentrated in densely populated built-up areas and related development and construction projects.

First of all, the protection of the ecological source should consider delimiting the core area, ensuring the habitat of the population and the intact natural landscape, and establishing the buffer zone outside the core area, so as to reduce the disturbance of human activities to the natural nature of the ecological source area as far as possible (19, 53). Second, ecological corridors are defined at different spatial and temporal scales to connect ecological sources and improve habitats by building forest road networks or building ecological bridges with local ecosystems. Finally, for the main supply areas of ESs such as nature reserves, forest parks, and reservoirs, it is necessary to develop tourism resources and eco-cultural industries to promote the realization of the value of ecological products.

**Figure 9:**
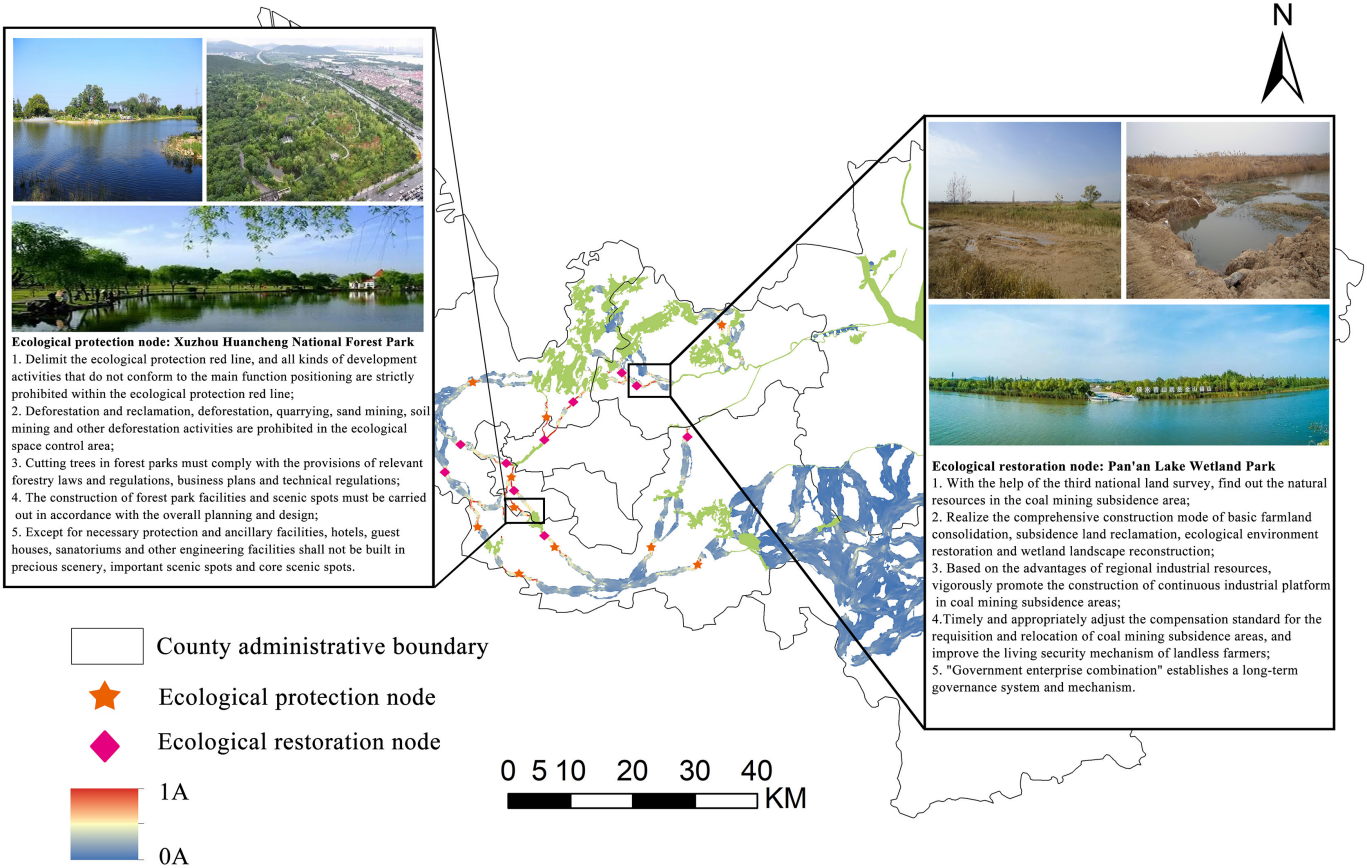
Two cases of key ecological protection/restoration areas in Xuzhou.

The identification of ESP can determine the overall situation of regional ecological protection/restoration. However, the ecological protection and restoration oriented to the implementation of the project need to determine the specific nodes. We selected two nodes that represent typical patterns of ecological protection and restoration (Figure 9).

For the ecological protection node, we chose Xuzhou Beltway National Forest. Xuzhou Ring City National Forest Park has a beautiful natural landscape and rich animal and plant resources. The region should, therefore, be protected through the establishment of regulatory measures. For example, through China's ecological red line policy, to limit the ecological red line of different types of development activities, including deforestation reclamation and deforestation quarrying, sand mining, and soil mining. For the ecological restoration node, we chose Pan'an Lake, Xuzhou. The area was once Xuzhou's largest coal-mining subsidence area, with an area of 12.6 km^2^. Such areas are typical of Xuzhou. On the one hand, the restoration of this kind of area should be done through the water system, construction of wetland ecological conservation area, and implementation of ecological interception and other ecological projects. On the other hand, a series of standards have been developed to regulate ecological restoration projects, such as “the technical standards for ecological restoration in North China Plain coal-mining subsidence areas.”

## 7. Conclusion and limitations

Ecological security is the manifestation of ESs, and the supply–demand of ESs is the bridge between ecological security and human wellbeing. The research has three possible contributions: (1) coupling the supply–demand theory of ESs with the construction of ESPs, (2) proposing a research framework for the ESPs of “supply–demand–corridor–node,” and (3) using the circuit theory model, the important ecological protection/restoration areas in the study area were identified. The results showed that the area of the supply source of ESs in Xuzhou City is 573.89 km^2^, accounting for 5.19% of the study area. The greatest ecological source patch covered 152.26 km^2^. The spatial distribution of 105 ecological corridors revealed that there were multiple and dense ecological corridors in the middle of the city, but few in the northwest and southeast. In all, 14 ecological protection areas were located primarily in the south of the urban area, and 10 ecological restoration areas were located primarily in the middle and north of the urban area, with a total area of 4.74 km^2^. The findings of this article would be useful in developing ESPs and determining important ecological protection/restoration areas in Xuzhou, China. This article shows that the supply and demand assessment of ESs not only integrates ecological processes and ecological functions but also fully considers the needs of economic society for ecology. The introduction of the perspective of supply—demand of ESs provides important theoretical guidance for the construction of ESPs.

There are still some limitations to this study. First of all, we did not consider the trade-offs and synergies between different ESs. Therefore, when we extract important ecological patches by superimposing a variety of ESs, duplication may occur (54, 55). Second, there are challenges in methods and standards when using ESs demand as the resistance surface. This article is only a preliminary exploration, which needs to be studied more systematically in the future. Finally, it is difficult to distinguish the supply and demand of ESs. For example, food production is not only the material supply of ESs but also the demand of human beings for ESs. More scientific index selection needs further research.

## Data availability statement

The original contributions presented in the study are included in the article/Supplementary material, further inquiries can be directed to the corresponding authors.

## Author contributions

ZW: writing—original draft and data curation. JZ: data curation and software. JC: editing and supervision. HG: supervision and writing—review and editing. JL: supervision. ML: writing—review and editing. All authors have read and agreed to the published version of the manuscript.
