# Supplementary material for: Determining the ecological security pattern and important ecological regions based on the supply–demand of ecosystem services: A case study of Xuzhou City, China
Source: Front Public Health. 2023 Feb 14;11:1087588. doi: 10.3389/fpubh.2023.1087588 (PMC9971807; doi:10.3389/fpubh.2023.1087588)
Supplement: Supplementary file 1 [file Data_Sheet_1.docx]

Supplementary Material

# Quantifying Ecosystem Services (ES)

## Water yield (WY)

In this paper, the water yield module of lnvest model is used to obtain the spatial distribution of water yield in the study area. Based on the principle of water balance, the module defines the water yield as the remaining water after the precipitation within the grid minus plant transpiration and surface evaporation, and assumes that the water yield of the grid unit finally reaches the watershed outlet through surface and underground runoff. The water yield is estimated through the parameters of precipitation, potential evapotranspiration, root system and soil depth, and corrected with the runoff data of the hydrological station at the outlet of the basin, so as to finally calculate the grid water yield of the basin. The main algorithms of the model are as follows:

$$\begin{aligned} &Y_{xj}=\left( 1-AET_{xj}/P_{x} \right)\times P_{x} \\ &\frac{AET(x)}{P(x)}=\frac{1+w_{x}R_{xj}}{1+w_{x}R_{xj}+1/R_{xj}} \end{aligned}$$

where $Y_{xj}$ represents the water yield of the x-th grid of land use type j (m^3^·hm^－2^); AET_xj_ represents the annual actual evapotranspiration of the x-th grid of land use type j (mm); $P_{x}$ represents the annual average precipitation of the x-th grid (mm); $R_{xj}$ represents the dryness index of the x-th grid of land use type j; $w_{x}$ represents the available water content of vegetation. The details of each coefficient are shown in **Table A1** of the supplementary data.

**Table A1.** Biophysical table in “water yield” module of InVEST model.

| **Land use type** | **Lucode** | **LULC_veg** | **root_depth** | **Kc** |
| --- | --- | --- | --- | --- |
| Farmland | 1 | 1 | 300 | 0.6 |
| Forest | 2 | 1 | 5000 | 1 |
| Grassland | 3 | 1 | 500 | 0.65 |
| Water body | 4 | 0 | 1 | 1 |
| Construction land | 5 | 0 | 1 | 0.3 |
| Bare land | 6 | 0 | 1 | 0.2 |

## Soil conservation (SC)

In this paper, the SDR module in the invest model is used to evaluate the soil conservation in the study area. According to the potential soil loss equation, the soil conservation (SD) is obtained by subtracting the potential soil erosion (RKLS) from the actual soil erosion (USLE). The formula is as follows:

$$\begin{aligned} &SC_{i}=RKLS_{i}-USLE_{i} \\ &RKLS_{i}=R_{i}\times K_{i}\times LS_{i} \\ &USLE_{i}=R_{i}\times K_{i}\times LS_{i}\times C_{i}\times P_{i} \end{aligned}$$

where $SC_{i}$ is soil conservation; $RKLS_{i}$ is the potential erosion amount; $USLE_{i}$ is the actual erosion amount; $R_{i}$ is Rainfall Erosivity Factor; $K_{i}$ is soil erodibility factor; $LS_{i}$ is the slope length factor; $C_{i}$ is the vegetation cover management factor; $P_{i}$ is the factor of water and soil conservation measures. The details of each coefficient are shown in **Table A2** of the supplementary data.

**Table A2.** Biophysical table in “sediment delivery ratio” module of InVEST model.

| **Land use type** | Lucode | usle_c | usle_p |
| --- | --- | --- | --- |
| Farmland | 1 | 0.5 | 0.4 |
| Forest | 2 | 0.06 | 1 |
| Grassland | 3 | 0.13 | 1 |
| Water body | 4 | 0 | 0 |
| Construction land | 5 | 0 | 0 |
| Bare land | 6 | 1 | 0 |

## Carbon storage (CS)

In this paper, the Carbon module in the InVEST model is used to calculate the carbon storage in the study area, and the carbon storage in the current landscape is estimated based on the land use type data in different periods and the storage in the corresponding four carbon pools. Calculation formula:

$$C_{\text{total }}=C_{\text{above }}+C_{\text{below }}+C_{\text{soil }}+C_{\text{dead }}$$

where C_tota_ is the total carbon storage; C_above_ is aboveground carbon storage; C_below_ refers to underground carbon storage; C_soil_ is soil carbon storage; C_dead_ is the carbon storage of dead organic matter. The unit is t·hm^－2^. The details of each coefficient are shown in **Table A3** of the supplementary data.

**Table A3.** Input data on carbon stored in each of the four fundamental pools for each land use type in the InVEST 3.8.0 model (Unit: Mg/ha).

| **Land use type** | C_above | C_below | C_soil | C_dead | lucode |
| --- | --- | --- | --- | --- | --- |
| Farmland | 0.54 | 0 | 8.67 | 0 | 1 |
| Forest | 2.65 | 0 | 11.3 | 0 | 2 |
| Grassland | 0.34 | 0 | 9.92 | 0 | 3 |
| Water body | 1.78 | 0 | 8.94 | 0 | 4 |
| Construction land | 0.48 | 0 | 8.1 | 0 | 5 |
| Bare land | 0 | 0 | 5.1 | 0 | 6 |

## Habitat quality (HQ)

In this paper, the Habitat Quality module of the InVEST model is used to evaluate the biodiversity of the study area and simulate the impact of human activities on the habitat. The greater the intensity of human activities, the greater the threat to the habitat, the lower the habitat quality and the lower the biodiversity level; On the contrary, the less human disturbance, the higher the habitat quality and the higher the biodiversity level. Combining the sensitivity of different land use types to threat factors and the intensity of external threats, the birth quality is calculated. The formula is as follows:

$$Q_{xj}=H_{j}\left[ 1-\left( \frac{D_{xj}^{z}}{D_{xj}^{z}+K^{z}} \right) \right]$$

where Q_xj_ represents the habitat quality of the x-th patch of land use type J; $D_{xj}^{z}$ represents the habitat stress degree of the x-th patch of land use type j; $H_{j}$ indicates the habitat suitability; $K^{z}$ is the semi saturation constant. Details of each factor are given in **Table A4** of the supplementary data.

**Table A4.** Biophysical table in “Habitat Quality” module of InVEST model.

| **Land use type** | HABITAT | L_built | L_cropland | L_bareland | L_road |
| --- | --- | --- | --- | --- | --- |
| Farmland | 0.25 | 0.5 | 0.3 | 0.4 | 0.2 |
| Forest | 0.95 | 0.9 | 0.8 | 0.4 | 0.3 |
| Grassland | 0.6 | 0.8 | 0.4 | 0.2 | 0.35 |
| Water body | 1 | 0.9 | 0.7 | 0.3 | 0.55 |
| Construction land | 0 | 0 | 0 | 0 | 0 |
| Bare land | 0.1 | 0.4 | 0.2 | 0 | 0.2 |
